# Positioning of aquatic animals based on time-of-arrival and random walk models using YAPS (Yet Another Positioning Solver)

**DOI:** 10.1038/s41598-017-14278-z

**Published:** 2017-10-30

**Authors:** Henrik Baktoft, Karl Øystein Gjelland, Finn Økland, Uffe Høgsbro Thygesen

**Affiliations:** 1 National Institute of Aquatic Resources, Technical University of Denmark, 8600 Silkeborg, Denmark; 2 Norwegian Institute of Nature Research, 9007 Tromsø, Norway; 3 Norwegian Institute of Nature Research, 7485 Trondheim, Norway; 4 National Institute of Aquatic Resources, Technical University of Denmark, 2800 Lyngby, Denmark

## Abstract

Aquatic positional telemetry offers vast opportunities to study *in vivo* behaviour of wild animals, but there is room for improvement in the data quality provided by current procedures for estimating positions. Here we present a novel positioning method called YAPS (Yet Another Positioning Solver), involving Maximum Likelihood analysis of a state-space model applied directly to time of arrival (TOA) data in combination with a movement model. YAPS avoids the sequential positioning-filtering-approach applied in alternative tools by using all available data in a single model, and offers better accuracy and error control. Feasibility and performance of YAPS was rigorously tested in a simulation study and by applying YAPS to data from an acoustic transmitter towed in a receiver array. Performance was compared to an alternative positioning model and proprietary software. The simulation study and field test revealed that YAPS performance was better and more consistent than alternatives. We conclude that YAPS outperformed the compared alternative methods, and that YAPS constitute a vast improvement to currently available positioning software in acoustic telemetry. Additionally, in contrast to vendor-supplied solutions, YAPS is transparent, flexible and can easily be adapted and extended for further improvements or to meet study specific requirements such as three-dimensional positioning.

## Introduction

Positional telemetry, i.e. the recording of animal positions over time, enables researchers to study the behaviour of aquatic animals in the wild. One approach to achieve this involves tagging animals with acoustic transmitters and monitor them using fixed position hydrophones. Subsequently, positions at the time of signal transmissions are calculated using tri- or multilateration based on differences in time of arrival at each hydrophone^1–3^. The obtained data can potentially be of high temporal and spatial resolution and can thus provide detailed information on the tagged animals’ locations^4–7^. This type of data offer vast opportunities to study the natural behaviour of individual animals in details not obtainable with other methodologies^8–12^.

Current manufacturers of positional telemetry systems provide either proprietary software or paid services for calculating positions based on data from deployed hydrophones. However, these solutions lack transparency and the underlying mathematical algorithms, models and assumptions are not publicly available. Generally, tracks of animal movement obtained through currently available systems need additional processing such as filtering^5,13^ to remove positional outliers or use of state-space models^14^ to accommodate observational errors.

Traditionally, estimation of positions of tagged aquatic animals from fixed position hydrophones have utilized time differences of arrival (TDOA) at detecting hydrophones^1–3,7,15,16^. With the TDOA approach, at least three hydrophones need to detect a given signal transmission in order to estimate a position in two dimensions; instances where only one or two hydrophones detect a transmission are discarded. Erroneous registration on just one hydrophone can potentially offset the estimated position from a few to several hundred meters even if the signal was detected correctly on a surplus of hydrophones (personal observation from simulation studies and field experiments). Since the occurrence of erroneous registrations have a probabilistic component, this can lead to a counterintuitive situation in which the accuracy and precision of a given calculated position deteriorates as the number of hydrophones detecting the signal increase^17^. Procedures eliminating information from assumedly invalid hydrophone registrations can be applied to reduce this effect in an over-determined system^16^. Our experience with several vendor-supplied positioning systems indicates that these apply some undescribed filtering algorithms. However, in data obtained using these systems positioning error may still be considerable even in over-determined hydrophone array configurations, including a relatively large proportion of positions with positional errors of several hundred meters (personal observation).

In this situation, we hypothesized that positioning could be improved by using the extra information in the time of arrival (TOA) of each ping at each hydrophone, rather than merely the time differences (TDOA), even if this information is imperfect. Moreover, that this information increases in value when combined with a model which describes the movement of the animal, so that information is shared optimally between subsequent signal transmissions. Finally, in broader terms, that the state of the art within acoustic positioning will advance more rapidly if algorithms are published and thus made available to the scientific community for scrutiny and further development.

Thus, we present YAPS (Yet Another Positioning Solver); a newly developed model for estimating aquatic animal positions combining signal time of arrival (TOA) at fixed position hydrophones with a random walk movement model. Our approach aims at being transparent and to be adaptable and extendable to fit study specific requirements. For instance, the present version of YAPS is intended for use in areas where the vertical dimension (i.e. depth) is negligible compared to the horizontal dimension (x and y). For use in areas where this is not true, YAPS can be extended to a 3D version in which the third dimension of positions is either estimated by the model or represented by data collected using transmitters with on-board pressure sensors thereby correcting estimation errors that would otherwise be introduced. The YAPS model was developed towards use with systems from a specific vendor (Lotek Wireless Inc., Newmarket, Ontario, Canada), offering transmitters with relatively stable burst intervals, which enables estimation of time of each transmission and subsequently using TOA as opposed to TDOA. However, YAPS is not vendor specific and is applicable to data obtained using other systems with similar characteristics, i.e. stable burst interval. By including a movement model fitted to raw TOA data, YAPS constraints the estimated positions to biologically plausible (under the movement model) outcomes. Moreover, YAPS utilises all available detections, even if number of hydrophones detecting a given signal is less than three. Additionally, by allowing residuals to be non-normally distributed, YAPS accommodates erroneous detections (e.g. multipath) that could otherwise result in severely biased position estimation. The model is interfaced using R^18^ and the position estimation is done using TMB (Template Model Builder^19^) to allow quick computation times.

To evaluate performance of YAPS, we applied the YAPS model and a standard TDOA model to two different datasets and compared results in terms of e.g. efficiency (i.e. number of estimated positions) and accuracy (deviation from true to estimated position): 1) data simulated from a random walk model and 2) data from a track with known trajectory obtained by combined movement of a differential global positioning system (DGPS) unit and transmitters in a hydrophone array setup in a river. The YAPS results from the latter dataset were also compared to results from a commercially available acoustic positioning software.

## Methods

### YAPS model formulation

The modelling follows the state space paradigm^20^ where we distinguish between first the process model, which describes the dynamics of the system and most importantly the *x*,*y*-location of the transmitter at time *t*, and next an observation model, which relates unobserved processes to data. The process model consists of the following stochastic processes: First, a model for the times *t(i)* of transmissions (measured in seconds since first detection) states that the interval between transmissions is a random walk:

1 $$t(i)-t(i-1)|t(i-1),t(i-2)\sim N(t(i-1)-t(i-2),{\sigma }_{bi}^{2})$$

here, our notation implies that the time *t(i)* of transmission number *i* is a random variable, specified in terms of its conditional distribution given *t(i-1)* and *t(i-2)*, which is a Gaussian with the specified mean and standard deviation. The transmission times are unobserved and estimated. This model component assumes that transmitter burst intervals can be modelled using a Gaussian distribution and allows some temporal variation that can be caused by temporally varying temperature affecting the frequency of the internal clock crystal.

Next, the model assumes an independent random walk for each co-ordinate (denoted *x* and *y*), where the variance of the displacement scales linearly with the time increment as in standard diffusion theory^21^:

2 $${\rm{x}}(t(i))|{\rm{x}}(t(i-1))\sim N({\rm{x}}(t(i-1)),{(2{D}_{xy}\ast (t(i)-t(i-1)))}^{0.5})$$

3 $$y(t(i))|y(t(i-1))\sim N(y(t(i-1)),{(2{D}_{xy}\ast (t(i)-t(i-1)))}^{0.5})$$

here, D_xy_ is the diffusivity, assumed to be identical in the x and y direction. The positions *x*,*y* (measured in meters) are unobserved and thus constitute an unobserved Markov processes, as is standard in the state space paradigm for time series analysis^20^.

To relate the time of transmissions with the time when the transmission reaches the receivers, we need the speed of sound *v*. Some variation in water temperature and density can occur even on small temporal and spatial scale and directly affects the speed of sound. We assume the speed to be a random walk:

4 $$v(t(i))|v(t(i-1))\sim N(v(t(i-1)),{(2{D}_{v}\ast (t(i)-t(i-1)))}^{0.5})$$

where D_v_ is the diffusivity of the velocity process. Note that we assume one value for the speed of sound at each transmission; we thus ignore differences in water temperature and density within each transmission as the sound wave propagates from the transmitter to the different receivers, and consider only differences between transmissions.

This concludes the process components in the model. Next, we relate the observed time *τ*_*i*,*H*_ when transmission *i* arrives at hydrophone *H*, to the transmission time *t(i)*. We compute Euclidian distances in two dimensions between the transmitter location *x*,*y(t(i))* at time *t(i)* and all hydrophone (*H*) positions (*x*_*H*_, *y*_*H*_):

5 $$D(H,t(i))={({({x}_{H}-x(t(i)))}^{2}+{({y}_{H}-y(t(i)))}^{2})}^{0.5}$$

Then, the predicted time of arrival (*μ*) at hydrophone (*H*) is:

6 $$\mu (H,i)=t(i)+D(H,t(i))/v(t(i))$$

We next define the residuals *E(H*,*i)* = *τ*_*i*,*H*_ − *μ(H*,*i)* between the predicted and observed time of arrival. We assume that this residual follows a mixture of two distributions: a Gaussian with mean 0, and a scaled t-distribution with three degrees of freedom. This distribution has well defined mean, variance and skewness, but displays heavy tails due to the t-distribution. Mixture ratio and scale parameters in the two distributions are estimated by the model.

### TDOA model formulation

Position estimation using a standard TDOA model is based on solving a set of hyperbolic equations each described by pairwise differences in time of arrival at three or more hydrophones^17^. The model assumes that difference in time of arrival of signal *i* emitted at time *t(i)* on hydrophones *Hn* and *Hm* follows a Gaussian distribution with mean *µ*_*TDOA*_ and variance ${\sigma }_{TDOA}^{2})$.

7 $$TDOA(Hn,Hm,t(i))\sim N({\mu }_{TDOA}(Hn,Hm,t(i)),{\sigma }_{TDOA}^{2})$$

In this, *µ*_*TDOA*_(*Hn*, *Hm*, *t*(*i*)) is defined by the linear predictor function:

8 $${\mu }_{TDOA}(Hn,Hm,t(i))={({({({x}_{Hn}-x(t(i)))}^{2}+{({y}_{Hn}-y(t(i)))}^{2})}^{0.5}-({({x}_{Hm}-x(t(i)))}^{2})+{({y}_{Hm}-y(t(i)))}^{2})}^{0.5}/v$$

in which *x(t(i))* and *y(t(i))* is location of the transmitter at time *i*, *x*_*Hn*_, *y*_*Hn*_, *x*_*Hm*_ and *y*_*Hm*_ are locations of the two focal hydrophones and *v* is speed of sound assumed to be constant.

### Computational analysis of the models

Both the YAPS and the TDOA models, as described in the previous, defines the joint distribution of all random variables in the model, both observed and unobserved, for given parameters. Following the approach in the Template Model Builder (TMB) framework^19^, the model is coded as a c++-file which evaluates the joint density. Unobserved random variables (i.e. *x*, *y*, *v* and *t* at each transmission) are automatically integrated out by TMB using the Laplace approximation, and parameters (fixed effects; i.e. *σ*_*bi*_, *D*_*xy*_, *D*_*v*_, residual standard deviation, the t-Gaussian mixture ratio and scale parameter for the t-distribution) are estimated using the Maximum Likelihood principle using a built-in optimizer (*nlminb()* from the package *stats*) in R^18^. Unobserved variables are estimated with the mean in their posterior distribution (again using the Laplace approximation). Uncertainties on parameter estimates are obtained from the Fisher information matrix, i.e. the curvature of the likelihood function, while uncertainties on random variables are obtained from the variance in the posterior distributions, still using the Laplace approximation. In summary, the model analysis is a standard Maximum Likelihood analysis of non-linear mixed-effects model, using TMB as the computational tool that automates the entire analysis.

### Simulation study

We simulated movement data *x*,*y(t)* as well as data for time of transmission *t(i)* and speed of sound *v(t(i))*. A simulated array consisting of five hydrophones positioned in the corners and centre of a 100*100 meter quadrant was used. Time of arrival matrices were established from this including Gaussian distributed noise to use as input to the model. This represents an optimal dataset in which all transmissions are registered as straight line detections with Gaussian distributed observation noise corresponding to hardware measurement error specific to the hydrophones. In total, 200 simulated tracks starting at random positions within the array and with 250 positions each were generated. We used fixed diffusivities for the simulated movement process (*D*_*xy*_ = 1.0 m^2^ * s^−1^) and speed of sound process (*D*_*v*_ = 0.01 m^2^ * s^−3^). To test the robustness of YAPS and to add realism to the simulation study, we introduced varying degrees of incomplete registrations by making detection on each hydrophone probabilistic; nine probabilities of missing detection (p(NA)) between 0.0 and 0.8 in increments of 0.1 were used. Additionally, to simulate detection of multipath propagated signals and random noise, henceforth collectively termed multipath, we introduced varying degrees of erroneous detections by offsetting correct time of arrival by random values corresponding to −100 to 100 meters assuming *v* = 1435 m*s^−1^. Six different probabilities of multipath (p(MP)) from 0.00 to 0.05 in increments of 0.01 were used. All 200 tracks were iteratively subjected to each combination of p(NA) and p(MP) five times and subsequently used as input to YAPS for estimating the simulated track. For comparison, all tracks and iterations were also positioned using a standard TDOA model with Gaussian distributed noise. Thus, each simulated track was iteratively estimated 9*6*5 = 270 times using each model.

In each model iteration a number of performance metrics were calculated for comparison of YAPS and the TDOA model: 1) relative number of estimated positions in each model run was determined. By definition, YAPS estimates a position for each transmission, whereas the TDOA model only estimates a 2D position if number of detecting hydrophones is three or more; 2) mean Euclidian distance between true and estimated positions; 3) relative deviation of estimated track length from true track length calculated as (length(estimated) – length(true))/length(true) * 100%. Additionally, the spatial component in position estimation error for both models was evaluated using Euclidian distance from estimated to true positions for full versions of all 200 simulated tracks (i.e. before introducing reduced detection probability (p(NA) = 0) and multipath (p(MP) = 0). Comparison with commercially available software was not possible.

### Applying YAPS to a known track

To further illustrate the utility of YAPS and to evaluate model performance under real study conditions, we applied YAPS to data from a setup in a Norwegian river covering a river stretch approximately 80 m wide and 400 m long. Twenty-seven hydrophones (Lotek 200 kHz WHS 3050, Lotek Wireless Inc., Newmarket, Ontario, Canada) were mounted to wall fixes (distance to surface 1.0 m) where applicable and otherwise mounted to iron poles erected from cross-like structures placed on the bottom (distance to surface range 0.7 m–3.1 m) and kept in place by heavy weights. Transmitters attached to a line (approximately one meter from surface) held vertical by weights were moved through the study site using a small inflatable row boat and true trajectory was established using a high precision differential gps-unit (logging frequency 1 Hz; Trimble Geo 7x cm edition running with the Norwegian Mapping Authority CPOS service) mounted above the transmitters (complete track is provided in results section). Median speed of the boat was 0.31 m * s^−1^. The study site was partly bounded by concrete walls, steel sluice gates, bedrock and large boulders, thus echoing, noise and multipath propagation was present. A miniature transmitter (Lotek, 200 kHz, model M-626, 7.5 × 17 mm, mass in air 1.0 g) with burst interval of approximately 2.01 seconds was used. Track duration within range of the hydrophone array was 108 minutes yielding 3244 transmissions. Obtained data were processed by the YAPS model, the TDOA model and the vendor provided software U-MAP (version 1.3.3, Lotek Wireless Inc., Newmarket, Ontario, Canada). U-MAP runs algorithms using the TDOA-principle, and may report twin positions in cases where the equations have two solutions. Depending on array configuration and quality of input data (e.g. amount of multipath propagated signals), TDOA models are prone to produce extreme outliers clearly outside the study area. These were filtered from the TDOA and U-MAP estimated tracks using distance to nearest hydrophone >500 m as criteria for removal. Optimization of post-processing routines was not the goal of this study, and no further filtering of TDOA or U-MAP estimated positions was applied. As the multipath induced temporal offset in the simulation study was limited to correspond to 100 m or less, the simulation study was not prone to such extreme outliers. Quality of estimated tracks was quantified by number of estimated positions and by calculating Euclidian distances from estimated positions to temporally closest true positions from the DGPS trajectory. To further test and demonstrate the utility of YAPS, we sub-sampled the data obtained from the described tow track to simulate a hydrophone array consisting of only eight hydrophones and applied YAPS on this data set.

All simulations and analyses (excluding U-MAP which is standalone software) were performed in R version 3.3.2^18^.

### Data Availability

Code and example data to run the YAPS model are available in the open github repository (https://github.com/baktoft/yaps_sciRep). Code to subsample the tow track, the hydrophone array and to simulate increased probability of missing detections are also provided.

## Results

### Simulation study

YAPS generally performed better than the TDOA model on all metrics. Additionally, performance of YAPS was less variable than performance of TDOA model, especially at increased p(NA) and p(MP).

#### Number of estimated positions

**Figure 1:**
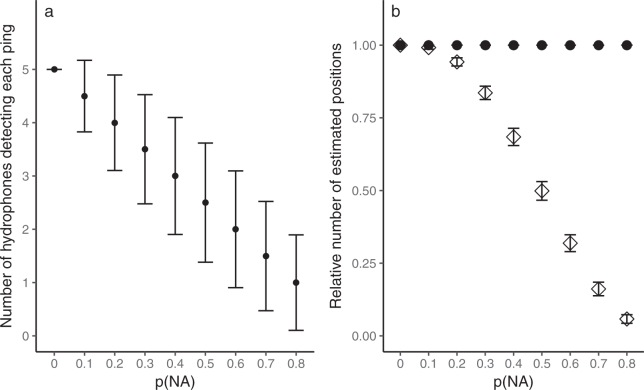
Probability of missing detections p(NA) affects relative number of estimated positions in the TDOA model, but not in YAPS. (**a**) Number of hydrophones detecting each transmission (mean values ± s.d.) as a function of probability of missing detections p(NA) in the 200 simulated tracks of 250 positions each. (**b**) Relative number of positions estimated (mean ± s.d.) by TDOA (◊) and YAPS (●) models in each iteration as a function of p(NA). Probability of multipath does not affect number of estimated positions, so only simulations where p(MP) = 0 were used. YAPS will by definition produce a fixed number of estimated positions equivalent to number of transmissions in the input data.

Whereas numbers of estimated positions from YAPS were fixed by number of transmissions in input data, number of estimated positions from TDOA model decreased as expected when p(NA) increased (Fig. 1).

#### Mean deviation from true to estimated positions

**Figure 2:**
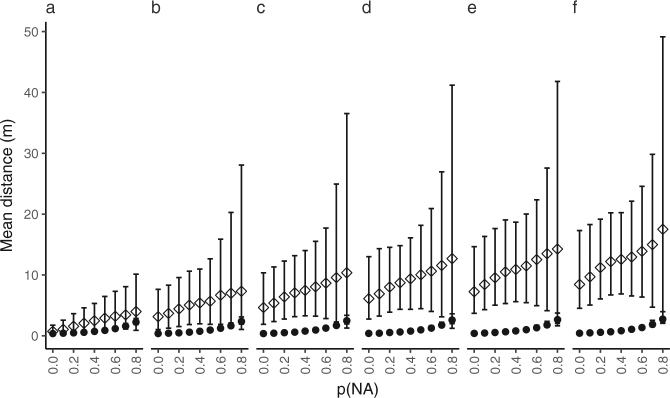
Effect of probability of missing detections (p(NA)) on model performance. Mean distance (±5^th^ and 95^th^ percentile) between true and estimated positions (TDOA (◊); YAPS (●)) for simulated tracks as a function of probability of missing detections (p(NA)). Each panel show results from one level of probability of multipath (p(MP)): (**a**) 0.00, (**b**) 0.01, (**c**) 0.02, (**d**) 0.03, (**e**) 0.04 and (**f**) 0.05.

Mean deviation from true to estimated positions increased in both YAPS and TDOA models as simulated p(NA) and p(MP) increased (Fig. 2). Best performance was achieved at optimal conditions (i.e. p(NA) = 0 and p(MP) = 0) under which mean deviation was 0.4 m for YAPS and 0.7 m for TDOA. Mean deviation increased to a maximum of 2.3 m for YAPS and 4.0 m for TDOA as p(NA) increased to 0.8. Fixating p(NA) = 0 and increasing p(MP) to 0.05 resulted in mean deviation of 0.4 m for YAPS and 8.5 m for TDOA. At the worst case scenario included in the simulation study (i.e. p(NA) = 0.8 and p(MP) = 0.05) mean deviation from true to estimated positions were 2.8 m for YAPS and 17.5 m for TDOA.

#### Relative error in track length estimation

**Figure 3:**
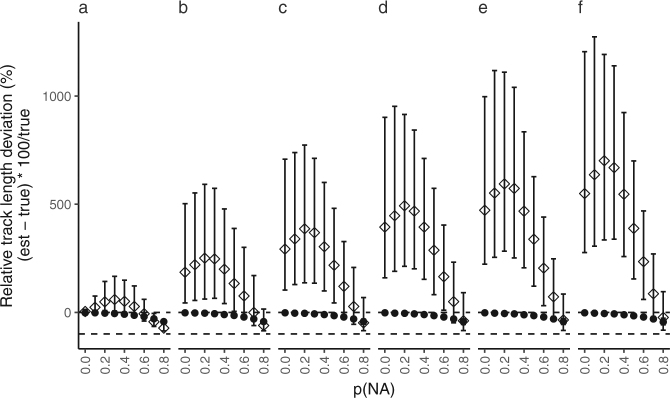
Effect of probability of missing detections (p(NA)) on relative track length. Mean relative track length differences between true and estimated track (±5^th^ and 95^th^ percentile; TDOA (◊); YAPS (●)). Negative values indicate that estimated tracks are shorter than true and *vice versa*. Each panel show results from level of probability of multipath (p(MP)): (**a**) 0.00, (**b**) 0.01, (**c**) 0.02, (**d**) 0.03, (**e**) 0.04 and (**f**) 0.05. Horizontal lines (- - -) indicating 0% and −100% are given for reference.

Deviation of estimated track length relative to true track length varied for both YAPS and TDOA models as p(NA) and p(MP) increased (Fig. 3). Both models performed best under optimal conditions (YAPS: −1.8% TDOA: 4.1%; positive and negative mean values indicate over- and under-estimation of track length, respectively). At increasing levels of p(NA) underestimation of track length from YAPS increased monotonically to maximum −43.0% as p(NA) = 0.8. Contrastingly, the TDOA model increased overestimation to maximum 59.5% at p(NA) = 0.5 followed by shift to underestimation of maximum −72.8% at p(NA) = 0.8. A slight rise in p(MP) to 0.01 resulted in 185.45% overestimation of track length in TDOA at p(NA) = 0, whereas track length estimated by YAPS remained relatively constant at −2.0%. At maximum simulated p(MP) = 0.05 mean overestimation by TDOA was 549.3% at p(NA) = 0; corresponding value for YAPS was −2.6%.

#### Spatial component of estimation error

**Figure 4:**
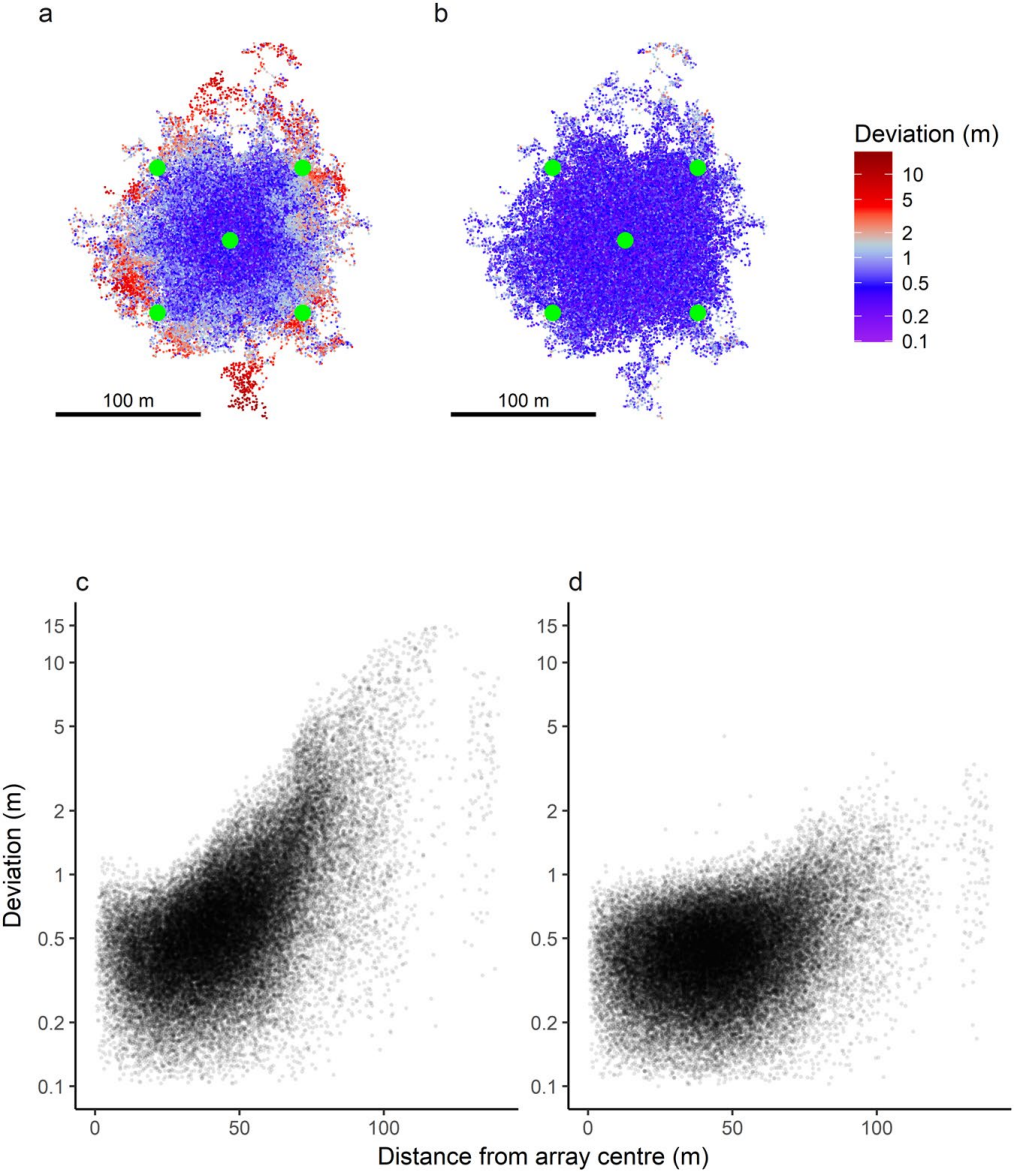
Spatial component of estimation error. Overall, both models performed well inside the hydrophone array, but estimation accuracy deteriorated more in the TDOA model as distance to array centre increased. (**a** and **b**) True trajectories of all 200 simulated tracks. Colours represent distance from estimated to true position for model TDOA (**a**) and YAPS (**b**); notice that colour scale is non-linear. Root mean square distances to true position was 1.21 for model TDOA and 0.44 for YAPS. The simulated hydrophone array () is indicated. (**c** and **d**) Distance from estimated to true position as a function of distance to array centre from model TDOA (**c**) and YAPS (**d**). Notice y-axis is log(y + 0.1)-transformed.

Performance variation in both YAPS and TDOA models included a spatial component with highest performance near the array centre (Fig. 4). Whereas YAPS performed relatively uniform inside the entire hydrophone array, performance of the TDOA model was more spatially variable both inside and outside the array. In locations outside the hydrophone array performance of the TDOA model decreased considerably more than YAPS.

### Applying YAPS to a known track

**Figure 5:**
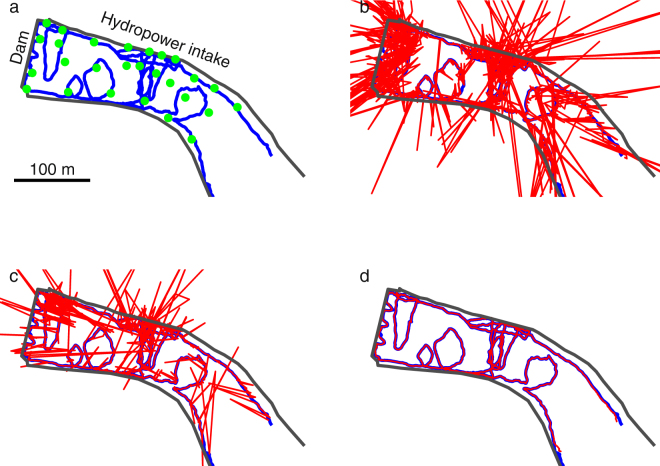
Performance comparison of positioning models applied on field data. Comparison of known track () obtained using DGPS positioned above the transmitter and tracks estimated () by (**b**) TDOA, (**c**) U-MAP and (**d**) YAPS. Track duration was 109 minutes yielding 3,244 transmissions of the transmitter. Physical boundary of the system is indicated by **—** and hydrophone positions () are indicated. Special attention (i.e. increased hydrophone density) was given to an area midway on the northern side as this constituted a hydro power intake.

Tracks estimated by YAPS, the TDOA model and U-MAP are shown in Fig. 5. Overall, all three positioning models revealed the major components of the true track, but the amount and severity of estimated erroneous positions clearly varied between models. As per model definition, number of estimated positions from YAPS was identical to number of transmissions in the input data (i.e. 3244) whereas TDOA model yielded 2892 (89%) positions (total number was 3017, but 125 was filtered out as gross outliers). Interestingly, number of positions estimated by U-MAP (2583 (80%)) was substantially lower than by TDOA model. Included in these were 39 twin position solutions (1.5% of the estimated positions). For the majority of the estimated track, YAPS corresponded closely to true track. Excluding instances where the transmitter was more than 50 m outside the array (only start and end of track) more than 87% of estimated positions were within 0.5 m of true position and more than 97% were within 1 m. For the TDOA model corresponding percentiles were 23% within 0.5 m and 49% within 1 m and for U-MAP 72% within 0.5 m and 82% within 1 m. YAPS performance was lowered in positions well outside the hydrophone array (i.e. the south-east corner), but still reflected the track well. Additionally, the area in front of the hydro power intake on the northern shore proved challenging for both the TDOA model and U-MAP, whereas YAPS performed well. The western end and the intake area were both bounded by steel gates and/or concrete walls, which undoubtedly induced a relatively large amount of echoing and multipath propagation. Computation time for YAPS was 28 minutes on a laptop with 16 GB RAM and an Intel Core i7-5600 processor (Intel, Santa Clara, California, USA) at 2.60 GHz.

**Figure 6:**
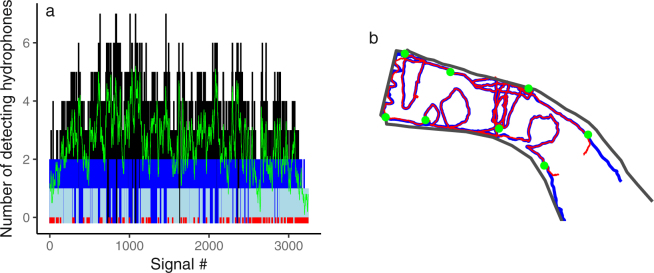
Sub-sampled tow track estimated by YAPS. The tow track data set was sub-sampled to simulate an eight-hydrophone array and YAPS was applied to estimate the track. (**a**) Number of hydrophones detecting each signal transmission. Signal transmissions detected by less than three hydrophones are highlighted using colours blue, light blue and red representing 2, 1 and 0 detecting hydrophones, respectively. () indicate running mean (window = 10) number of detecting hydrophones. (**b**) Track estimated by YAPS () overlain known track obtained using DGPS (). Approximately 50% of estimated positions were with 0.5 m of true position and 80% were within 1 m.

Results from sub-sampling the tow-track to simulate an eight-hydrophone array and applying YAPS on these data are shown in Fig. 6. Mean number of hydrophones detecting each transmission was 2.5 and percentage of transmissions detected by three or more hydrophones was 49%. As defined by the model, number of estimated positions was identical to number of transmission in the input data (i.e. 3244). The track estimated by YAPS resembled the true track well as more than 50% of estimated positions were within 0.5 m of true position and more than 80% were within 1 m.

## Discussion

In this study, we have demonstrated and tested the application of the newly developed YAPS model to estimate movement trajectories of aquatic animals equipped with acoustic transmitters monitored by fixed position hydrophone arrays. The simulation study and application to a known track obtained under field conditions demonstrated YAPS as a suitable tool to estimate such movement trajectories. Performance of YAPS was superior to a standard TDOA model and also better than the vendor supplied software U-MAP. Performance by U-MAP was considerably better than the TDOA model although number of estimated positions was lower. This, and the fact that U-MAP occasionally uses less than the available number of detections for position estimation in an over determined system, indicate that U-MAP employs more than a simple TDOA model to improve quality of output data.

The power of YAPS originates from the (to our knowledge) unique unified approach of applying a combination of a state space model describing animal movement and a positioning model directly to TOA data. By doing this, the animal movement model is constraining the probability space of estimated positions to biologically plausible (under the movement model) outcomes. Combined with the Gaussian and t mixture distribution for model residuals, this effectively accommodates TOA data suffering from multipath propagation, which otherwise can be detrimental for data quality. Additionally, TOA-based positioning as employed in YAPS has benefits over position estimation based on TDOA. For instance, from theory it is known that TOA based estimation is less affected by hydrophone array configuration^17^. Furthermore, as evidenced by the simulation study and the sub-sampled data set simulating an eight-hydrophone array, TOA based position estimation and the combination of an animal movement model and a positioning model enables position estimation for transmissions where number of hydrophones detecting the transmission is less than three, albeit with increased uncertainty of the estimate. YAPS is therefore able to utilize information otherwise lost when using the TDOA approach. It should be noted that YAPS needs a certain minimum amount of data to function properly, but rigorous tests of this lower threshold is outside the scope of the present study. However, preliminary trials performed using subsets of the tow track presented here, indicate that data sets consisting of ten or more consecutive signal transmissions detected on average on 1.9 hydrophones can be enough to obtained good results.

**Figure 7:**
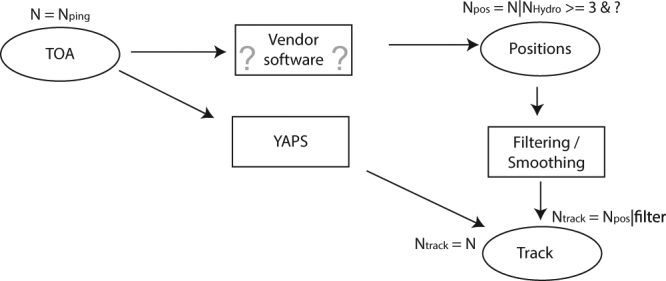
Conceptual model showing data processing pathways using YAPS and vendor supplied software. While YAPS utilizes all available hydrophone registrations to estimate positions for all transmissions, data volume is progressively condensed and reduced in the alternative pathway leading to loss of information and ultimately less estimated positions in the final track. Additionally, YAPS provides direct estimation of movement model parameters using all available data. Moreover, as indicated by the question marks in the figure, available vendor supplied software are proprietary and the underlying models and filter algorithms are not accessible to the user. Therefore, exact model formulation and potential filtering criteria are unknown.

The unified multilevel modelling in YAPS contrasts alternative data processing pathways (Fig. 7), which, in a step-wise approach apply filtering and/or smoothing models to improve data quality (e.g. biological filters typically based on swimming speed or vendor provided position quality metrics) to positions previously estimated using vendor supplied software comparable to U-MAP (e.g.^2,13,22^).

We consider the YAPS model presented here as a solid base that can be adapted and extended for further improvement and to meet study specific needs. For instance, a third component to the random walk model describing animal movements can easily be added to facilitate acoustic based tracking of tagged animals in three dimensions.

A previous study found that positioning based on random walk models work well even in situations, where the animals being tracked behave according to other movement models such as piecewise constant velocity and Levy flight^22,23^. Additionally, the fact that YAPS performed well in estimating the tow track (which did not perform a random walk), indicates that YAPS is robust in estimating tracks arisen from other movement models. Nevertheless, the movement model component of YAPS can be extended to more complex models such as correlated random walk models or the Ornstein-Uhlenbeck process which previously have been used to estimate animal movement trajectories^24,25^. Depending on vendor, acoustic transmitters might be designed to transmit at irregular time intervals. At present the YAPS model is capable of positioning such tags, but the performance in comparison to alternative solutions has not been thoroughly assessed and is beyond the scope of the present study.

The improvement in attainable quality of aquatic animal tracking represented by YAPS was made possible by the fact that the hardware manufacturer (Lotek Wireless Inc.) enabled researchers to extract high resolution raw data from the hydrophones (i.e. TOA data). We advocate that such possibility to access raw data should be *de facto* standard from all manufacturers providing telemetry hardware to the scientific community.
